# Temporal Reproducibility of Fracture Interpretation in Forensic Radiography: A Multispecialty Comparison of Physicians and Vision Language Models Including Fracture Subtype Description

**DOI:** 10.3390/tomography12080113

**Published:** 2026-08-12

**Authors:** Halit Canberk Aydogan, Adem Köksal, Ali Aygün, Hacer Yaşar Teke, Feyza Nur Çatalbaş, İbrahim Çaltekin

**Affiliations:** 1 Department of Forensic Medicine, Ordu University Training and Research Hospital, Ordu 52200, Türkiye; 2 Department of Emergency Medicine, Ordu University Training and Research Hospital, Ordu 52200, Türkiye

**Keywords:** forensic radiography, fracture interpretation, temporal reproducibility, vision language models, fracture subtype classification

## Abstract

Background: Accurate fracture interpretation on plain radiographs is critical for both trauma care and medico-legal decision-making, where reproducibility is as important as point accuracy. Although vision language models (VLMs) have shown promising diagnostic performance, their temporal stability in forensic radiography remains unclear. Methods: We analyzed 300 forensic radiographs (150 fracture-positive, 150 fracture-negative) from six long bones, independently evaluated by three emergency medicine physicians, three forensic medicine physicians, and three VLMs (ChatGPT-5.2, Gemini 3 Pro, Claude Sonnet 4.5) using an identical task format. Assessments included fracture presence and structured fracture subtype description (bone, morphology, displacement). VLM evaluations were repeated after one month under identical conditions. Results: Physician accuracy ranged from 79.7% to 98.7%. Emergency physicians reached the higher median sensitivity (92.0% against 76.7%), while specificity among the forensic readers was the more tightly clustered (median 92.0%, range 87.3–100.0%). ChatGPT-5.2 was the most accurate model (83.0%; sensitivity 70.0%, specificity 96.0%), followed by Gemini 3 Pro (77.7%), whereas Claude Sonnet 4.5 reached only 46.3% because of an extreme false-positive tendency (specificity 11.3%). Over one month, accuracy changed by −5.2, −4.1 and +8.6 percentage points, but these net figures concealed considerable case-level movement: within-model agreement ranged from near chance to substantial (mean Cohen κ 0.131 to 0.622), and the F1-score of Claude Sonnet 4.5 fell by 13.1 points despite its higher accuracy. Subtype descriptions were frequently correct once a fracture had been detected, but end-to-end subtype accuracy remained low. Conclusions: Current vision language models demonstrate encouraging diagnostic performance; however, their temporal reproducibility remains inadequate for independent medico-legal fracture interpretation. These findings highlight that reproducibility, in addition to diagnostic accuracy, should be considered a core benchmark when evaluating VLMs for high-stakes clinical and forensic use. Larger multicenter studies using independent external datasets are needed before forensic application is considered.

## Simple Summary

Vision language models (VLMs) are attracting increasing interest for medical image interpretation, but their consistency over time has been evaluated only to a limited extent. In this study, we compared three vision language models with experienced physicians for radiographic fracture and fracture subtype interpretation and examined the reproducibility of their assessments after one month. Our findings suggest that temporal reproducibility, together with diagnostic performance, may provide additional insight when evaluating the potential role of vision language models in clinical and medico-legal radiographic interpretation. These observations come from a single center and should be regarded as preliminary.

## 1. Introduction

Traumatic musculoskeletal injuries constitute a pervasive global health burden, with bone fractures representing a primary cause of morbidity, long-term functional disability, and substantial healthcare resource utilization worldwide [1]. In the high-pressure and time-constrained environment of emergency departments, plain radiography remains the cornerstone of initial diagnostic triage due to its cost-effectiveness, speed, and widespread accessibility. However, the interpretation of radiographic images is inherently susceptible to human perceptual errors, particularly under conditions of clinician fatigue, cognitive overload, or limited subspecialty experience [2]. Missed fractures account for a substantial proportion of diagnostic discrepancies in the emergency department [3,4] and may lead to delayed treatment, nonunion, and permanent impairment, while generating significant medico-legal liability for healthcare providers [5,6].

In response to these diagnostic challenges, the integration of artificial intelligence (AI) into medical imaging has transitioned from theoretical exploration to tangible clinical application over the past decade [7]. Deep learning algorithms, particularly convolutional neural networks (CNNs), have emerged as the dominant paradigm for automated fracture detection [8]. Systematic reviews and meta-analyses have demonstrated that AI-assisted diagnostic systems can achieve performance comparable to expert radiologists, especially in detecting fractures of the appendicular skeleton [9]. Anatomically focused syntheses report similar behavior for the ankle and foot [10], and explainable AI has extended deep learning beyond fracture detection to other foot radiographic diagnoses such as pes planus, improving model transparency and supporting clinical interpretation [11]. Nevertheless, these traditional AI models are primarily designed for narrow, task-specific objectives, such as binary fracture classification, and often lack the capacity to integrate contextual clinical information or to generate structured, human-like reasoning that aligns with real-world medical documentation and decision-making processes [12,13].

More recently, the field has undergone a paradigm shift with the emergence of foundation models that process visual and textual input jointly, including GPT-4, Gemini, and Claude [14]. Terminology in this literature is inconsistent, and vision language model, multimodal large language model, and large language model are often used interchangeably. Throughout this article we use vision language model (VLM) for a model that accepts an image together with a text prompt, which is the class of system evaluated here, and reserve large language model (LLM) for text-only systems described in the cited work. Unlike earlier unimodal architectures, these models can simulate aspects of the cognitive workflow employed by physicians during image interpretation [15]. Early validation studies suggest that they are capable not only of detecting radiographic abnormalities but also of generating structured reports and context-aware interpretative outputs [16]. Quantitative assessments across anatomical regions nevertheless report uneven diagnostic performance and a marked tendency to over-report abnormalities on normal images [17,18]. The deployment of generative AI in high-stakes medical settings therefore raises critical concerns regarding reliability, consistency, and reproducibility [19].

Unlike deterministic algorithms, these models are inherently stochastic and may produce variable outputs in response to identical inputs, increasing the risk of hallucinated or internally inconsistent interpretations [20]. This limitation is particularly consequential in forensic medicine, where the differentiation between acute injury, chronic changes, or post-traumatic artifacts directly informs legal responsibility, causal attribution, and compensation-related decisions [21]. Consequently, there is a pressing need to assess whether current models can satisfy the stringent evidentiary standards required for medico-legal fracture interpretation, rather than solely meeting clinical performance benchmarks [22,23].

Three gaps persist in this literature. Comparisons have been predominantly single-specialty, usually radiologists against an automated system, and forensic medicine has been largely absent despite the central role of fracture interpretation in judicial processes. Performance has almost always been reported cross-sectionally, so it is not known whether a model returns the same decision for the same image at a later date. And subtype-level output, meaning morphology and displacement, which is what a medico-legal report requires, has rarely been evaluated apart from binary detection.

This study therefore compares emergency medicine physicians, forensic medicine specialists, and three vision language models on an identical set of plain radiographs, with three aims: (i) to quantify fracture detection performance and inter-reader agreement; (ii) to measure temporal reproducibility by repeating the whole assessment after one month under identical prompting; and (iii) to evaluate structured fracture subtype description separately from binary detection.

## 2. Methods

### 2.1. Study Sample and Case Characteristics

A total of 300 plain radiographic images were included in the analysis, comprising 150 fracture-positive and 150 fracture-negative cases. The images represented six long-bone regions (humerus, radius, ulna, femur, tibia, and fibula). For each bone, a total of 50 images were evaluated, consisting of 25 fracture and 25 non-fracture cases. All cases were forensic in nature and had been reported as medico-legal cases in the emergency department.

The images were obtained from patients who presented to a tertiary-care emergency department due to forensic-related trauma between 1 January 2020 and 30 June 2025, were formally reported as medico-legal cases during the emergency department process, and additionally underwent forensic medicine examination. All radiographic images were acquired as part of routine emergency department trauma evaluation. All images were reviewed in their original clinical format without post-processing or resolution enhancement. Each radiographic case was treated as an independent assessment unit for all reader and model evaluations. For each case, date of birth, age, date of forensic medicine examination, sex, and injury origin were recorded, and descriptive statistics for these variables were reported in the study. The archive contained adult cases only, and no patient under 18 years of age was included.

This study was approved by an institutional non-interventional research ethics committee (Approval No. 2025/173, decision date: 9 May 2025). The study was conducted in accordance with the Declaration of Helsinki and relevant national regulations.

### 2.2. Readers, Blinding, and Assessment Workflow

Radiographs were independently evaluated by two physician reader groups and three vision language models. Physician readers consisted of three forensic medicine specialists and three emergency medicine specialists, each with a minimum of five years of clinical experience in fracture assessment within forensic or emergency medicine practice. Physicians reviewed the images independently and were blinded to all clinical information other than the radiographic images.

The physician assessment process was standardized to be content-equivalent to the task definition applied to the models. Physicians were instructed to provide a fracture presence/absence decision and, when a fracture was identified, a structured assessment including the involved bone, fracture morphology, and displacement status.

Physicians were invited to participate via email, and study materials were sent to five forensic medicine specialists and six emergency medicine specialists. Physicians were asked to submit their evaluations online to the research team and were given two days to respond. During the assessment period conducted between 14 December 2025 and 16 December 2025, two forensic medicine specialists and three emergency medicine specialists who did not respond within the specified timeframe were excluded from the study. The final physician sample therefore consisted of three forensic medicine specialists and three emergency medicine specialists.

### 2.3. Vision Language Models and Inference Framework

Vision language model assessments were conducted using ChatGPT-5.2 (OpenAI), Gemini 3 Pro (Google DeepMind), and Claude Sonnet 4.5 (Anthropic). Each system was queried in the consumer version current at the time of testing: GPT-5.2, made publicly available on 11 December 2025; Gemini 3 Pro, released on 18 November 2025; and Claude Sonnet 4.5, released on 29 September 2025. All three remained the current release of their respective product lines throughout the study window, since the successor versions (GPT-5.3, Gemini 3.1 Pro and Claude Sonnet 4.6) appeared only in February 2026, after the second assessment had been completed. This study is reported in accordance with the TRIPOD + AI guideline [24], and the completed checklist is provided as Supplementary Table S1. Reporting was additionally checked against consensus recommendations for studies of language models in radiology [25]. To ensure procedural standardization, the same task instruction and output format were applied across all models (standardized prompt; Supplementary Table S2).

The usage paradigm employed in this study was zero-shot inference. No example-based prompting, contextual training, or intermediate reasoning guidance (chain-of-thought strategies) was applied. In addition, no fine-tuning, retrieval-augmented generation (RAG), few-shot prompting, or any other model adaptation techniques were used. All models were accessed through their publicly available web-based user interfaces rather than via application programming interfaces (APIs) and were operated in their commercially available versions. No user-adjustable sampling parameters were available within the employed interface workflows; therefore, all outputs reflect default generation settings.

Each case was submitted to the models as an independent assessment unit, and all model outputs were recorded verbatim. A representative screenshot illustrating the standardized prompt, radiographic image submission interface, and output format is provided in Supplementary Figure S1.

### 2.4. Temporal Stability Assessment

Model evaluations were conducted at two time points: an initial assessment (T0) and a repeat assessment after one month (T1). T0 evaluations were performed between 14–16 December 2025, and T1 evaluations were conducted between 14–16 January 2026.

One month was chosen a priori as a compromise. It is long enough to span the kind of backend update that vendors deploy without notice on consumer platforms, so it reflects the drift an ordinary user would meet rather than an artificial same-session repetition, yet short enough for the image set and the reference standard to remain untouched. It also approximates the interval that separates the initial emergency department reading from the forensic medicine review of the same images in the medico-legal pathway.

The one-month repeat assessment was applied exclusively to the models; no repeat assessment was performed for physician readers, since the question concerned the stability of automated output rather than human intra-reader variability. At both time points, the same image set, identical standardized prompt, and identical output requirements were used. For each evaluation, models were instructed to provide (i) a fracture presence decision and, when a fracture was reported, (ii) a structured fracture subtype description including bone, morphology, and displacement. Because explicit version locking and build-level documentation are not supported in consumer-facing interfaces, silent backend model updates cannot be fully excluded and are therefore considered an inherent component of real-world temporal variability.

### 2.5. Reference Standard for Fracture Classification

Fracture status was determined using a predefined reference standard incorporating orthopedic consultation and clinical follow-up information documented in the medical records.

Fracture-positive cases were defined as those confirmed through concordant assessments by emergency medicine, orthopedic surgery, and radiology services, with corresponding forensic medicine documentation consistent with the confirmed fracture diagnosis, and clinical management documented in the medical record.

Fracture-negative cases were defined using a combined clinical, radiological, and follow-up based approach. A case was classified as fracture-negative only when all of the following criteria were met: (i) radiological evaluation of the plain radiographic images demonstrated no acute fracture findings; (ii) clinical evaluations by emergency medicine and forensic medicine did not support a fracture diagnosis; and (iii) clinical follow-up demonstrated no subsequent fracture diagnosis, with no record of computed tomography, surgical intervention, or orthopedic treatment requirement.

### 2.6. Outcomes and Definitions

A two-layer outcome framework was applied:

Primary outcome: Fracture detection performance, defined as agreement between the fracture presence decision for each case and the reference standard.

Secondary outcome: Accuracy of fracture subtype description in reference-standard fracture-positive cases. When a fracture was reported, the following structured attributes were evaluated: (i) involved bone, (ii) fracture morphology, (iii) displacement status.

Fracture subtype performance was quantified using three predefined indices:

- End-to-end exact match (pipeline exact match): the proportion of all reference-standard fracture-positive cases in which both the fracture decision and the morphology and displacement information were simultaneously correct.
- Conditional exact match: the proportion of correctly detected fractures for which both morphology and displacement were accurately reported.
- Over-explanation in non-fracture cases: the proportion of reference-standard fracture-negative cases in which a fracture subtype description was provided despite the absence of fracture. For physician readers, fracture subtype fields were completed only when a fracture was identified; thus, over-explanation reflects instances where subtype information was provided despite a fracture-negative reference standard.

One-month temporal reproducibility was evaluated only for model outputs and assessed for both fracture detection decisions and fracture subtype descriptions.

### 2.7. Statistical Analysis

Statistical analyses were performed to compare fracture detection performance between physician readers and vision language models. Diagnostic performance was summarized using accuracy, sensitivity, specificity, positive predictive value, and F1-score. Performance metrics were calculated separately for each reader and model and, where appropriate, summarized descriptively at the group level.

Agreement among physician readers and among models was evaluated using kappa statistics. Temporal reproducibility was assessed by comparing T0 and T1 evaluations using within-model agreement measures. Paired analyses were applied to examine changes in fracture detection decisions and fracture subtype classifications between T0 and T1. Change is reported as Δ = T1 − T0 in percentage points, and discordant pairs are given separately in each direction so that case-level movement can be distinguished from net change.

All statistical analyses were performed using IBM SPSS Statistics for Windows, version 25.0 (IBM Corp., Armonk, NY, USA).

## 3. Results

### 3.1. Study Population and Injury Etiology

A total of 300 forensic cases were included. The mean age was 34.8 ± 13.2 years (median, 33; range, 18–82), and 71.3% were male (*n* = 214). Assault was the most frequent injury etiology (31.7%). Traffic-related injuries accounted for 42.7% overall (*n* = 128), distributed as in-vehicle (14.0%), out-of-vehicle (15.3%), and motorcycle accidents (13.3%). Occupational accidents constituted 14.7% of cases, whereas falls (4.3%), firearm injuries (4.7%), and sharp-object injuries (1.7%) were less frequent. Detailed demographics and injury etiology are provided in Supplementary Table S3.

### 3.2. Overall Fracture Detection Performance

Overall fracture detection performance of physician readers and vision language models is summarized in Table 1. Among forensic medicine physicians, accuracy ranged from 82.0% to 98.7%, while among emergency medicine physicians it ranged from 79.7% to 98.3% (median accuracies: 84.3% and 90.3%, respectively).

At baseline (T0), ChatGPT-5.2 achieved an accuracy of 83.0% with sensitivity 70.0% and specificity 96.0%. Gemini 3 Pro yielded an accuracy of 77.7% (sensitivity 62.7%, specificity 92.7%). In contrast, Claude Sonnet 4.5 showed markedly reduced accuracy (46.3%), driven by extremely low specificity (11.3%) despite relatively high sensitivity (81.3%), consistent with a false-positive-prone decision profile. ChatGPT-5.2 and Gemini 3 Pro were therefore specificity-weighted rather than balanced: the sensitivity of both fell below that of every physician reader, while the specificity of ChatGPT-5.2 (96.0%) exceeded the median of both physician groups.

### 3.3. Bone-Specific Fracture Detection Performance and Inter-Reader Agreement

Bone-specific fracture detection performance is presented in Table 2. For each long bone, physician results are reported as median (min–max) accuracy across three readers per specialty, whereas model results correspond to baseline evaluation (T0).

Across bones, forensic medicine physicians showed median accuracies ranging from 82.0% (radius) to 96.0% (humerus), and emergency medicine physicians from 90.0% (ulna) to 98.0% (humerus). Wider between-reader ranges were observed particularly for the radius and fibula. Model performance varied substantially by bone: ChatGPT-5.2 ranged from 76.0% to 94.0% (femur) and Gemini 3 Pro from 68.0% (radius) to 84.0% (fibula). Claude Sonnet 4.5 was the weakest model at five of the six bones (16.0–52.0%); the ulna was the single exception (78.0%), where its binary accuracy matched the other two models even though its agreement between the two time points at that bone was no better than chance (κ = 0.000).

Inter-reader agreement for binary fracture decisions is summarized in Supplementary Table S4. Agreement was highest for the humerus in both physician groups (Fleiss’ κ: 0.921 for forensic medicine; 0.817 for emergency medicine) and lowest for the radius (κ: 0.538 and 0.520, respectively). Agreement among the models was low across bones (κ ranging from −0.169 to 0.289), with confidence intervals frequently including zero.

### 3.4. One-Month Temporal Stability of Fracture Decisions

One-month temporal reproducibility of model fracture decisions is summarized in Table 3. ChatGPT-5.2 lost 5.2 percentage points of accuracy (83.0% to 77.8%) while its F1-score barely moved (80.5% to 80.2%), and it retained the highest within-model agreement (mean Cohen’s κ 0.622). Gemini 3 Pro behaved similarly on a lower baseline (accuracy 77.7% to 73.6%, Δ −4.1; F1-score 73.7% to 73.4%, Δ −0.3; mean κ 0.450). Claude Sonnet 4.5 was the outlier: accuracy rose from 46.3% to 54.9% (Δ +8.6) while its F1-score fell from 60.2% to 47.1% (Δ −13.1), with the lowest agreement of the three models (mean κ 0.131). The divergence between the two metrics points to a redistribution of errors rather than genuine improvement.

Aggregate figures understated how much moved at the level of individual cases. Discordant pairs between the two time points numbered 12 for ChatGPT-5.2 (8 correct to incorrect, 4 in the opposite direction), 19 for Gemini 3 Pro (11 and 8), and 32 for Claude Sonnet 4.5 (13 and 19). Because these transitions were roughly symmetric in direction, McNemar’s test was non-significant for all three models (*p* > 0.05), which indicates the absence of a systematic directional shift rather than the absence of instability. Bone-level agreement is given in Supplementary Table S5.

### 3.5. Fracture Subtype Classification Performance (Morphology + Displacement) and Over-Explanation

Fracture subtype performance (morphology plus displacement) is summarized in Supplementary Table S6. Across bones, physician readers achieved higher end-to-end exact-match (“pipeline”) rates than the models, with the highest pipeline values observed for femur and humerus (up to 48.0% and 40.0%, respectively). In contrast, model pipeline exact-match remained low across bones at T0 (typically 0.0–24.0%). Nevertheless, conditional exact-match among true-positive detections was moderate-to-high for ChatGPT-5.2 and Gemini 3 Pro in several bones (e.g., conditional exact-match reaching 83.3–100.0% in ulna, femur, and tibia), suggesting that when a fracture was correctly detected, subtype descriptions were often internally consistent. Over-explanation in fracture-negative cases was minimal for ChatGPT-5.2 and Gemini 3 Pro (generally 0.0–8.0%), whereas Claude Sonnet 4.5 showed substantially higher over-explanation (e.g., humerus 38.5% and femur 36.0% at T0), aligning with its false-positive-prone profile.

### 3.6. One-Month Stability of Subtype Performance

Temporal stability of subtype metrics over one month (T0 → T1) is presented in Supplementary Table S7. Across models, T1 reassessment produced modest, bone-dependent changes in pipeline and conditional exact-match rates without a uniform directional trend across bones. Over-explanation generally decreased for Claude Sonnet 4.5 (e.g., humerus 38.5% → 20.0%; femur 36.0% → 24.0%), while remaining low and largely unchanged for ChatGPT-5.2 and Gemini 3 Pro (0.0% to 4.0% at T1). Overall, subtype-level outputs showed heterogeneous temporal behavior across bones, and model-specific tendencies, particularly the over-calling pattern of Claude Sonnet 4.5, persisted at follow-up.

## 4. Discussion

### 4.1. Performance Discrepancies and Decision Profiles

This study revealed significant disparities in diagnostic performance and decision-making strategies between physician groups and vision language models. Emergency medicine (EM) physicians read with higher sensitivity, which fits their mandate to avoid missed diagnoses in acute trauma and matches the literature identifying missed fractures as a leading source of diagnostic error in the emergency department [26]. Specificity separated the two specialties differently, in dispersion rather than in central tendency: the forensic medicine (FM) readers clustered tightly, consistent with the medico-legal need for evidentiary certainty [27], whereas the EM readers spread across more than thirty percentage points and included the lowest specificity recorded in the study. Among the models, ChatGPT-5.2 came closest to the physician readers on accuracy, and it did so with a specificity-weighted profile rather than a balanced one: its specificity exceeded the median of both physician groups while its sensitivity fell below that of every human reader. Its errors therefore resembled those of the forensic readers, a pattern consistent with the deep learning benchmarks reported in recent meta-analyses [28]. Claude Sonnet 4.5 inverted the profile in the most damaging direction, reporting a fracture on roughly nine of every ten normal radiographs. The spread between these two, from conservative to hypersensitive, contrasts sharply with the consistent, role-driven strategies of the human experts.

### 4.2. Professional Roles and Liability in Decision Thresholds

The decision thresholds observed between EM and FM physicians reflect the influence of professional liability and workflow context on diagnostic behavior. One EM reader paired the lowest specificity in the study, 67.3%, with a sensitivity of 92.0%, a trade-off that mitigates the malpractice risk attached to a missed fracture in trauma care; no forensic reader fell below 87.3%. While AI systems have shown potential to reduce human error rates [29], our findings suggest they currently lack the context-aware risk calibration inherent to trained physicians. Legal frameworks in Europe and the US emphasize that liability remains with the human operator, necessitating AI tools that align with these professional standards [30,31]. The variability of Gemini 3 Pro and the excessive false positives of Claude Sonnet 4.5 indicate that off-the-shelf models are not yet calibrated for the high-stakes environment of medico-legal decision-making, where the cost of a false positive differs significantly from clinical triage [32,33].

### 4.3. Strategic Differences Among Vision Language Models

Our findings highlight determining strategic differences in how various model architectures approach radiographic interpretation. ChatGPT-5.2 achieved the highest F1-score (80.5%) among the models. This aligns with recent scoping reviews suggesting that advanced GPT models are increasingly capable of handling complex medical reasoning tasks, although validation remains inconsistent [34]. In contrast, Claude Sonnet 4.5’s behavior was characterized by an aggressive “false-positive weighted” strategy. This behavior resembles the “over-call” tendency often seen in early computer-aided detection (CAD) systems, which can lead to “alert fatigue” and reduced clinical efficiency [35]. These distinct profiles suggest that “one-size-fits-all” prompting is insufficient, and model-specific calibration is essential to align AI strategies with specific clinical or forensic goals [36].

### 4.4. Medico-Legal Reliability and Accuracy

Although select models like ChatGPT-5.2 achieved “acceptable” accuracy levels comparable to human baselines reported in systematic reviews, they failed to meet the stringent reliability standards required for medico-legal practice. High accuracy alone is insufficient for forensic admissibility; consistency, explainability, and reproducibility are paramount. Physicians in our study demonstrated high internal consistency and clear adherence to professional standards. In contrast, the models exhibited stochastic behavior and, in the case of Claude Sonnet 4.5, frequent “hallucinations” of fractures in normal images. The same failure has been documented in general radiology, where a vision language model fabricated findings and misidentified detail on images it could not interpret [37]. Together with wider critiques of the “black box” nature of generative models [38], this currently precludes their use as independent expert witnesses or definitive diagnostic tools in court.

### 4.5. Temporal Instability and Its Practical Significance

A critical limitation identified in this study is the non-deterministic nature of model outputs over time, a flaw rarely observed in human expert practice. What makes it consequential is visible only at the case level. Claude Sonnet 4.5 is the clearest illustration: its accuracy rose over the month while its F1-score fell by more than thirteen points and about one radiograph in ten was decided differently. Aggregate movement of a few percentage points can therefore coexist with a substantial reshuffling of individual cases.

This matters because forensic conclusions are issued case by case. A claimant or a defendant is affected by the reading of one specific radiograph, and a system that reverses that reading between two examinations of the same image cannot support a stable evidentiary conclusion, whatever its group accuracy. Bone-level agreement makes the point concrete: within-model κ fell to 0.000 for the radius, ulna, and tibia for Claude Sonnet 4.5 and to 0.114 for the ulna for Gemini 3 Pro, values indicating agreement no better than chance. Comparable instability and drift have been reported in other longitudinal evaluations of generative models [39,40], and reproducibility has recently been proposed as a core reporting requirement for measurement tasks performed by these systems [41].

Two features of the deployment environment bear on how this should be read. Because the models were queried through public consumer interfaces, undocumented backend updates cannot be separated from sampling variation without pinned API versions; a forensic expert consulting the same platform would face exactly that opacity, so we regard it as a property of the systems in use rather than an artifact of our design. What can be excluded is a change of version. GPT-5.2, Gemini 3 Pro and Claude Sonnet 4.5 were each the current release of their product line at both time points, their successors appearing only in February 2026, three weeks or more after the second assessment. The instability therefore arose within a single nominal release, which is precisely the situation a user would assume to be stable. The three systems did differ in maturity at first testing, GPT-5.2 having been public for three days against 26 and 76 days for the other two, so post-release tuning remains one candidate mechanism within that release.

### 4.6. Fracture Morphology and Subtype Consistency

While the models struggled with the primary task of detection stability, they showed moderate promise in describing fracture characteristics conditional upon correct detection. For correctly identified fractures, ChatGPT-5.2 and Gemini 3 Pro achieved high conditional exact-match rates for morphology and displacement in specific bones. This suggests that the “language” component of these models can effectively structure reports once the visual feature is detected, a capability supported by recent studies on automated report generation [42,43]. However, this utility is severely limited by upstream detection errors. If the model cannot reliably decide whether a fracture exists, its ability to describe what it sees becomes clinically irrelevant for screening purposes [44].

### 4.7. Anatomical Variability in Performance

Consistent with previous deep learning literature, our results showed that anatomical complexity significantly impacts performance for both humans and AI. Performance decrements were observed in anatomically complex regions like the radius and fibula compared to the femur or humerus. The ulna is the instructive case: Claude Sonnet 4.5 combined its highest binary accuracy there with its weakest temporal agreement, so anatomical difficulty and reproducibility do not track one another. This mirrors findings from specific anatomical studies where AI performance varies widely based on bone superimposition and image quality [45,46,47,48]. The models’ precipitous performance drop suggests that current multimodal vision encoders still struggle more than human experts with “noise” and fine-grained feature extraction in complex anatomical backgrounds [49].

### 4.8. Patient Age and Forensic Radiographic Interpretation

Age is recorded in every forensic file, so its likely effect on these results deserves comment. Our cohort was adult throughout (18 to 82 years, mean 34.8 ± 13.2), and most cases fell between the second and fifth decades. The immediate consequence is that pediatric fracture morphology is absent. Greenstick, torus, and physeal injuries dominate childhood long-bone trauma and are exactly the patterns in which subtle cortical discontinuity, open growth plates, and secondary ossification centers are confused most easily. Neither our accuracy estimates nor our agreement estimates transfer to minors, which is a real restriction given that age estimation and child abuse assessment are routine forensic radiographic tasks.

Older patients were present but sparse. Reduced bone mineral density, degenerative change, healed fractures, and orthopedic hardware all alter cortical appearance and would be expected to lower specificity, since each offers a plausible substrate for a false-positive call, and to lower sensitivity for low-energy insufficiency fractures; the over-calling of Claude Sonnet 4.5 would plausibly worsen in such images. Site-dependent and population-dependent variation of this kind is documented for deep learning fracture detection [10]. We did not pre-specify an age-stratified analysis, and the design does not support one reliably: with 50 images per bone, further partitioning by age band leaves strata too small for stable estimation. These expectations are therefore hypotheses, and testing them requires a prospective dataset with adequate pediatric and geriatric representation in which age is treated as a pre-specified effect modifier.

### 4.9. Clinical and Forensic Utility

The findings suggest a dichotomy in potential utility: while ChatGPT-5.2 and Gemini 3 Pro show limited potential as clinical decision support tools (CDSS), none are currently viable for independent forensic use. In a clinical CDSS role, the high sensitivity of certain models could serve as a “second pair of eyes” to reduce missed fractures in the ED, provided a physician verifies the output [50]. However, for forensic applications such as independent expert testimony or definitive injury certification, the lack of temporal consistency and the high rate of false positives are fatal flaws. The inability to guarantee reproducible results undermines the legal principle of reliability required for expert evidence [51].

The comparison itself carries one qualification. Our physicians read the images without any clinical information, a deliberate choice that gave human and machine readers identical input. Practice does not work that way: an emergency physician knows the mechanism of injury and the point of maximal tenderness, and a forensic physician has the case file and the examination findings. Withholding that context almost certainly depressed physician performance below what routine work would produce, so our physician figures are best read as an image-only floor. The comparison is fair in its inputs but understates, rather than overstates, the distance between human and model performance in practice.

### 4.10. Requirements for Future AI in Forensics

These findings collectively indicate that for AI to be suitable for forensic use, future development must prioritize deterministic outputs, temporal consistency, and rigorous, context-specific validation. Generic “accuracy” metrics are insufficient; models must demonstrate stability (identical output for identical input over time) and calibration to the high-specificity requirements of legal standards [52,53]. Furthermore, validation must move beyond general datasets to include “stress tests” on difficult medico-legal cases (e.g., subtle fractures, post-mortem artifacts) [54].

External validation is the most urgent of these. A single-center cohort shares acquisition protocols, detector hardware and case mix, so estimates obtained under those conditions are known to run optimistic against multi-institutional data. Until independent datasets from other hospitals, with different equipment and different forensic case profiles, have been tested, none of the figures reported here can be treated as generalizable.

## 5. Limitations

This study is based on a single-center, retrospective forensic cohort, and no independent external dataset was available for validation, which may limit the generalizability of the findings to non-forensic trauma populations. The balanced distribution of fracture-positive and fracture-negative cases does not fully reflect real-world prevalence but was intended to standardize performance comparisons; predictive values should therefore not be transferred to settings with a different fracture prevalence.

The cohort was adult throughout, and no age-stratified analysis was pre-specified, for the reasons set out in Section 4.8.

Physician assessments were intentionally conducted without additional clinical context to ensure methodological comparability with the model evaluations; therefore, real-world diagnostic decision-making processes may not be fully represented. Temporal reproducibility was assessed only for the models, and direct comparison with human intra-reader stability was not performed. Finally, all models were evaluated using their commercially available versions, and performance may differ under regulated or institution-specific deployment conditions. Most diagnostic metrics are reported as point estimates, with confidence intervals given principally for agreement statistics.

## 6. Conclusions

In this multi-specialty forensic radiography cohort, emergency and forensic physicians demonstrated role-consistent decision thresholds, whereas the three vision language models showed marked heterogeneity and appreciable instability over time. The most accurate of them approached the physician readers on point accuracy, yet none reached the reproducibility that medico-legal fracture interpretation implicitly requires. Temporal stability therefore emerges as a critical and under-evaluated dimension, and accuracy alone is not an adequate basis for judging these systems.

These findings are preliminary. They come from one center, one image archive, one prompting strategy, and a single one-month interval, and they should not be extrapolated to other settings, to pediatric radiographs, or to future model versions. Larger multicenter studies with independent external datasets, version-controlled model access, and repeated longitudinal measurement are needed before any conclusion about forensic suitability can be drawn. On present evidence, vision language models should be confined to supervised supportive roles rather than independent medico-legal decision-making.

## Figures and Tables

**Table 1:** Overall fracture detection performance of physician readers and vision language models.

Reader/Model	TP	TN	FP	FN	Accuracy (%)	Sensitivity (%)	Specificity (%)	PPV (%)	F1-Score (%)
Forensic Medicine Physician 1	115	131	19	35	82.0	76.7	87.3	85.8	81.0
Forensic Medicine Physician 2	146	150	0	4	98.7	97.3	100.0	100.0	98.6
Forensic Medicine Physician 3	115	138	12	35	84.3	76.7	92.0	90.6	83.0
Emergency Medicine Physician 1	138	101	49	12	79.7	92.0	67.3	73.8	81.9
Emergency Medicine Physician 2	145	150	0	5	98.3	96.7	100.0	100.0	98.3
Emergency Medicine Physician 3	129	142	8	21	90.3	86.0	94.7	94.2	89.9
ChatGPT-5.2 (T0)	105	144	6	45	83.0	70.0	96.0	94.6	80.5
Gemini 3 Pro (T0)	94	139	11	56	77.7	62.7	92.7	89.5	73.7
Claude Sonnet 4.5 (T0)	122	17	133	28	46.3	81.3	11.3	47.8	60.2

TP, true positive; TN, true negative; FP, false positive; FN, false negative; PPV, positive predictive value. Sensitivity = TP/(TP + FN); Specificity = TN/(TN + FP); PPV = TP/(TP + FP); Accuracy = (TP + TN)/total cases; F1-score = harmonic mean of precision and recall. Each reader and model assessed 150 fracture-positive and 150 fracture-negative cases. Model values correspond to the baseline evaluation (T0).

**Table 2:** Bone-specific fracture detection performance.

Bone	Forensic Medicine Physicians–Accuracy % (Median [Min–Max])	Emergency Medicine Physicians–Accuracy % (Median [Min–Max])	ChatGPT-5.2 Accuracy %	Gemini 3 Pro Accuracy %	Claude Sonnet 4.5 Accuracy %
Humerus	96.0 (94.0–100.0)	98.0 (88.0–100.0)	88.0	82.0	42.0
Radius	82.0 (76.0–100.0)	94.0 (80.0–100.0)	76.0	68.0	50.0
Ulna	88.0 (84.0–96.0)	90.0 (72.0–94.0)	76.0	74.0	78.0
Femur	94.0 (86.0–100.0)	96.0 (92.0–100.0)	94.0	82.0	40.0
Tibia	86.0 (78.0–100.0)	96.0 (78.0–100.0)	88.0	76.0	52.0
Fibula	92.0 (76.0–98.0)	94.0 (80.0–96.0)	76.0	84.0	16.0

Values for physician groups are reported as median (min–max) across three readers per specialty. Each bone subgroup included 25 fracture-positive and 25 fracture-negative cases (*n* = 50). Model results correspond to the baseline evaluation (T0).

**Table 3:** One-month temporal stability of vision language model fracture decisions (T0 vs. T1).

Model	T0 Accuracy (%)	T1 Accuracy (%)	ΔAccuracy (pp)	T0 F1 (%)	T1 F1 (%)	ΔF1 (pp)	Cohen’s κ (T0 ↔ T1) Mean Across Bones; Range	b (T0 Correct → T1 Incorrect)	c (T0 Incorrect → T1 Correct)	McNemar *p*
ChatGPT-5.2	83.0	77.8	−5.2	80.5	80.2	−0.3	0.622 (0.273–0.883)	8	4	0.3877
Gemini 3 Pro	77.7	73.6	−4.1	73.7	73.4	−0.3	0.450 (0.114–0.746)	11	8	0.6476
Claude Sonnet 4.5	46.3	54.9	+8.6	60.2	47.1	−13.1	0.131 (0.000–0.571)	13	19	0.3771

Cohen’s κ values quantify within-model temporal agreement between baseline (T0) and 1-month re-evaluation (T1) for binary fracture decisions (fracture present/absent) under identical prompting and identical image sets. Analyses were performed separately for each long bone (per bone *n* = 50; 25 fracture-positive and 25 fracture-negative cases). κ values close to 0 indicate agreement no better than chance, whereas higher values indicate increasing temporal stability. T0 values are identical to those in Table 1. Δ = T1 − T0, in percentage points. b, cases correct at T0 and incorrect at T1; c, cases incorrect at T0 and correct at T1.

## Data Availability

The datasets are not publicly available because they contain anonymized forensic radiographic images subject to legal, ethical, and institutional restrictions.

## Supplementary Materials

The following supporting information can be downloaded at: https://www.mdpi.com/article/10.3390/tomography12080113/s1, Table S1: TRIPOD + AI checklist compliance; Table S2: Standardized prompt used for vision language model evaluation; Table S3: Demographic characteristics and injury etiology of the study population; Table S4: Bone-specific inter-reader agreement (Fleiss’ κ, 95% CI); Table S5: Bone-level temporal agreement of vision language model fracture decisions; Table S6: Bone-level fracture subtype performance (morphology + displacement) at T0 and T1; Table S7: One-month temporal stability of fracture subtype classification metrics across bones; Figure S1: Representative screenshot illustrating the standardized prompt, radiographic image submission interface, and output format used for vision language model evaluations. The same prompt structure and output requirements were applied across all models and both time points (T0 and T1). The image file is supplied separately.
