# Supplementary material for: Temporal Reproducibility of Fracture Interpretation in Forensic Radiography: A Multispecialty Comparison of Physicians and Vision Language Models Including Fracture Subtype Description
Source: Tomography. 2026 Aug 12;12(8):113. doi: 10.3390/tomography12080113 (PMC13517668; doi:10.3390/tomography12080113)

Supplementary Materials

Temporal Reproducibility of Fracture Interpretation in Forensic Radiography: A Multispecialty Comparison of Physicians and Vision Language Models Including Fracture Subtype Description

Supplementary Table S1. TRIPOD+AI checklist compliance

Section / Topic	Item	Dev / Eval	Checklist Requirement	Completed?	Manuscript Location / Notes
Title	1	E	Identify study as developing/evaluating prediction model	Yes	Title clearly indicates evaluation; forensic radiographs; fracture interpretation and reproducibility focus
Abstract	2	E	TRIPOD+AI abstract structure	Yes	Abstract includes background, methods, results and conclusions; data, readers/models; repeated evaluation (T0/T1); key results
Introduction	3a	E	Context + rationale	Yes	Introduction (fracture burden; ED errors; AI/CNN; vision language models)
	3b	E	Target population + intended use	Yes	Introduction (forensic medico-legal pathway; intended users: EM/FM; medico-legal implications)
	3c	E	Health inequalities	N/A	No health inequality or fairness analysis planned for this forensic radiograph evaluation dataset
Objectives	4	E	Study objectives	Yes	End of Introduction (three numbered aims: multi-specialty comparison; temporal stability T0/T1; subtype description)
Data	5a	E	Data sources	Yes	Methods 2.1 (single-center forensic ED radiographs; routine care imaging)
	5b	E	Dates of data collection	Yes	Methods 2.1 (1 Jan 2020–30 Jun 2025); temporal testing dates in 2.4
Participants	6a	E	Study setting	Yes	Methods 2.1 (tertiary-care ED; single center; forensic cases)
	6b	E	Eligibility criteria	Yes	Methods 2.1 (forensic-related trauma; medico-legal reporting; forensic examination; adult-only archive)
	6c	E	Treatment details	N/A	Interpretation/evaluation study; treatment pathways not analyzed
Data Preparation	7	E	Pre-processing + quality checks	Partial	Methods 2.1 (original clinical format; no post-processing applied)
Outcome	8a	E	Outcome definition	Yes	Methods 2.5–2.6 (reference standard; primary fracture detection; secondary subtype metrics)
	8b	E	Outcome assessor characteristics	Partial	Methods 2.5 describes reference standard via orthopedic consult + concordant EM/orthopedics/radiology documentation
	8c	E	Blinding of outcome	N/A	Retrospective reference standard based on existing records
Predictors	9a	D	Initial predictors	N/A	No model development performed
	9b	E	Predictor definitions	Yes	Methods 2.2–2.3 (input = radiograph image; standardized prompt/output format)
	9c	E	Predictor assessor characteristics	Yes	Methods 2.2 (physicians ≥5y experience; blinded to clinical info).
Sample Size	10	E	Sample size rationale	Partial	Sample size was determined by a balanced design across six long bones (25 fracture-positive and 25 fracture-negative cases per bone; total n=300). No formal power or precision-based sample size calculation was performed.
Missing Data	11	E	Handling of missing data	Yes	Complete-case analysis; all included cases were analyzed
Analytical Methods	12a	E	Data use for evaluation	Yes	Methods 2.7 (performance metrics per reader/model; bone-level analyses; agreement; paired T0/T1 analyses; Δ defined as T1 − T0)
	12b	D	Predictor handling in development	N/A	No model development
	12c	D;E	Model type + rationale	Yes	Methods 2.3 (off-the-shelf ChatGPT/Gemini/Claude; web UI; no fine-tuning)
	12d	D;E	Heterogeneity across clusters	N/A	Single-center design; no clustering
	12e	D;E	Performance metrics + plots	Yes	Methods 2.7 + Results Tables 1–3; Supp Tables S4–S7
	12f	E	Model updating	N/A	No recalibration or updating performed
	12g	E	How predictions generated	Yes	Methods 2.3 (standardized prompt; web UI; screenshot Supp Fig S1; no API)

Section / Topic	Item	Dev / Eval	Checklist Requirement	Completed?	Manuscript Location / Notes
Class Imbalance	13	E	Methods used + rationale	N/A	Dataset was intentionally balanced by design (150 fracture-positive / 150 fracture-negative; 25/25 per bone); therefore, no class imbalance handling methods were required.
Fairness	14	E	Fairness assessment	N/A	Study not designed or powered for subgroup fairness analysis; absence of a pre-specified age-stratified analysis is stated in Limitations
Model Output	15	E	Output definition	Yes	Methods 2.6 and standardized prompt (binary fracture decision; structured subtype fields)
Training vs Evaluation	16	D;E	Dataset differences	N/A	Only evaluation dataset used (no development or training dataset in this study)
Ethics	17	E	IRB approval	Yes	Methods 2.1 (approval No. 2025/173; decision date; Helsinki; regs)
Open Science	18a	E	Funding	Yes	Funding statement provided; no external funding
	18b	E	Conflicts of interest	Yes	Conflict of Interest statement provided
	18c	E	Study protocol	No	Protocol not publicly available; held by the institutional ethics committee
	18d	E	Registration	No	Study not prospectively registered
	18e	E	Data sharing	Yes	Data Availability Statement provided; data restricted by legal, ethical and institutional rules
	18f	E	Code sharing	N/A	No custom analysis code developed; models queried via public web interfaces; statistics in SPSS
PPI	19	E	Patient/public involvement	No	No patient or public involvement in this retrospective image-based evaluation
Results	20a	E	Participant flow	Partial	Results 3.1 + Methods 2.1 give totals; no flow diagram
	20b	E	Participant characteristics	Yes	Results 3.1 + Supp Table S3
	20c	E	Comparison of development vs evaluation data	N/A	No development dataset
Model Development	21	D;E	Participant numbers per analysis	N/A	No model development
Model Specification	22	D	Full model details	N/A	External proprietary vision language models; version locking not possible (Methods 2.4)
Model Performance	23a	E	Performance + confidence intervals	Partial	Performance metrics (accuracy, sensitivity, specificity, F1-score) are reported as point estimates in Tables 1–3. Confidence intervals were provided primarily for agreement statistics (κ) in Supplementary Table S4; this is stated in Limitations.
	23b	E	Heterogeneity in clusters	N/A	Single-center; no multi-site variation
Model Updating	24	E	Updated model	N/A	No updating performed
Discussion	25	E	Interpretation + fairness	Yes	Discussion 4.1–4.10
	26	E	Limitations	Yes	Section 5 (Limitations)
	27a	E	Handling poor-quality inputs	Partial	Discussion 4.7–4.8 (anatomical complexity; image noise; age-related bone quality)
	27b	E	Required user expertise	Yes	Discussion 4.9 (supervised supportive role; physician verification)
	27c	E	Next research steps	Yes	Discussion 4.10 (external multicenter validation; pinned model versions; age stratification)

Dev, development; Eval, evaluation; D, development item; E, evaluation item; N/A, not applicable to this study design.

Supplementary Table S2. Standardized prompt used for vision language model evaluation

You are shown one or more plain radiographic images of a long bone from the same case.<br>Task:<br>1) Determine whether a fracture is present.<br>- Answer strictly as:<br>Fracture present = 1<br>Fracture absent = 0<br>2) If a fracture is present, describe the fracture using standard orthopedic terminology.<br>Your description must include:<br>- Bone involved<br>- Fracture morphology (transverse, oblique, spiral, comminuted, segmental, impacted, avulsion)<br>- Displacement status (displaced or non-displaced)<br>Rules:<br>- Do not provide clinical recommendations.<br>- Do not provide differential diagnoses.<br>- Do not speculate beyond the visible images.<br>- Base your answer only on visual information in the images.<br>- If no fracture is present, do not provide any description.<br>Output format:<br>Fracture presence: [0 or 1]<br>Fracture description: [only if fracture = 1]

Supplementary Table S3. Demographic characteristics and injury etiology of the study population

Section	Category	n	%	Summary
Demographics	Age (years)	–	–	Mean ± SD: 34.8 ± 13.2; Median (min–max): 33 (18–82)
	Sex			
	Male	214	71.3	–
	Female	86	28.7	–
Injury etiology	Assault	95	31.7	–
	In-vehicle traffic accident	42	14.0	–
	Out-of-vehicle traffic accident	46	15.3	–
	Motorcycle accident	40	13.3	–
	Occupational accident	44	14.7	–
	Fall	13	4.3	–
	Firearm injury	14	4.7	–
	Sharp-object injury	5	1.7	–
	Sports-related injury	1	0.3	–
	Total	300	100.0	–

The archive was adult-only; no case younger than 18 years was included.

Supplementary Table S4. Bone-specific inter-reader agreement (Fleiss' κ, 95% CI)

Bone	Forensic medicine physicians (κ [95% CI])	Emergency medicine physicians (κ [95% CI])	Vision language models (κ [95% CI])
Humerus	0.921 [0.817, 1.000]	0.817 [0.682, 0.922]	0.041 [−0.234, 0.292]
Radius	0.538 [0.345, 0.706]	0.520 [0.329, 0.680]	−0.115 [−0.403, 0.086]
Ulna	0.679 [0.516, 0.837]	0.568 [0.389, 0.729]	0.220 [−0.026, 0.371]
Femur	0.785 [0.643, 0.918]	0.760 [0.598, 0.893]	0.180 [−0.156, 0.464]
Tibia	0.643 [0.447, 0.803]	0.653 [0.489, 0.804]	0.289 [−0.200, 0.747]
Fibula	0.589 [0.398, 0.754]	0.672 [0.498, 0.830]	−0.169 [−0.403, 0.100]

Fleiss' κ was used to quantify within-group agreement for binary fracture decisions (fracture present/absent). Values are presented with 95% confidence intervals (CI). Negative κ values may occur when agreement is worse than chance; CIs including 0 indicate uncertainty in agreement beyond chance.

Supplementary Table S5. Bone-level temporal agreement of vision language model fracture decisions

Bone	ChatGPT-5.2 κ (T0↔T1)	Gemini 3 Pro κ (T0↔T1)	Claude Sonnet 4.5 κ (T0↔T1)
Humerus	0.883	0.638	0.066
Radius	0.273	0.471	0.000
Ulna	0.690	0.114	0.000
Femur	0.754	0.746	0.151
Tibia	0.467	0.333	0.000
Fibula	0.667	0.400	0.571

Cohen's κ values quantify within-model temporal agreement between baseline (T0) and 1-month re-evaluation (T1) for binary fracture decisions (fracture present/absent) under identical prompting and image sets (per bone: n=50; 25 fracture-positive, 25 fracture-negative). κ≈0 indicates agreement no better than chance.

Supplementary Table S6. Bone-level fracture subtype performance (morphology + displacement) at T0 and T1

Group	Reader/Model	Bone	T0 Pipeline	T0 Conditional	T0 Over-explain	T1 Pipeline	T1 Conditional	T1 Over-explain
Forensic medicine	Forensic Medicine Physician 1	Humerus	40.0	43.5	3.8	N/A	N/A	N/A
Forensic medicine	Forensic Medicine Physician 1	Radius	16.0	21.1	16.0	N/A	N/A	N/A
Forensic medicine	Forensic Medicine Physician 1	Ulna	24.0	31.6	20.0	N/A	N/A	N/A
Forensic medicine	Forensic Medicine Physician 1	Femur	36.0	47.4	4.0	N/A	N/A	N/A
Forensic medicine	Forensic Medicine Physician 1	Tibia	16.0	22.2	16.0	N/A	N/A	N/A
Forensic medicine	Forensic Medicine Physician 1	Fibula	20.0	29.4	16.7	N/A	N/A	N/A
Forensic medicine	Forensic Medicine Physician 2	Humerus	32.0	34.8	0.0	N/A	N/A	N/A
Forensic medicine	Forensic Medicine Physician 2	Radius	12.0	12.0	0.0	N/A	N/A	N/A
Forensic medicine	Forensic Medicine Physician 2	Ulna	32.0	32.0	0.0	N/A	N/A	N/A
Forensic medicine	Forensic Medicine Physician 2	Femur	36.0	36.0	0.0	N/A	N/A	N/A
Forensic medicine	Forensic Medicine Physician 2	Tibia	16.0	16.7	0.0	N/A	N/A	N/A
Forensic medicine	Forensic Medicine Physician 2	Fibula	16.0	16.7	0.0	N/A	N/A	N/A
Forensic medicine	Forensic Medicine Physician 3	Humerus	32.0	33.3	3.8	N/A	N/A	N/A
Forensic medicine	Forensic Medicine Physician 3	Radius	12.0	20.0	8.0	N/A	N/A	N/A
Forensic medicine	Forensic Medicine Physician 3	Ulna	28.0	35.0	12.0	N/A	N/A	N/A
Forensic medicine	Forensic Medicine Physician 3	Femur	48.0	52.2	4.0	N/A	N/A	N/A
Forensic medicine	Forensic Medicine Physician 3	Tibia	8.0	13.3	4.0	N/A	N/A	N/A
Forensic medicine	Forensic Medicine Physician 3	Fibula	12.0	16.7	16.7	N/A	N/A	N/A
Emergency medicine	Emergency Medicine Physician 1	Humerus	28.0	29.2	19.2	N/A	N/A	N/A
Emergency medicine	Emergency Medicine Physician 1	Radius	12.0	15.0	44.0	N/A	N/A	N/A
Emergency medicine	Emergency Medicine Physician 1	Ulna	20.0	21.7	48.0	N/A	N/A	N/A
Emergency medicine	Emergency Medicine Physician 1	Femur	44.0	44.0	20.0	N/A	N/A	N/A
Emergency medicine	Emergency Medicine Physician 1	Tibia	16.0	16.7	32.0	N/A	N/A	N/A
Emergency medicine	Emergency Medicine Physician 1	Fibula	20.0	22.7	33.3	N/A	N/A	N/A
Emergency medicine	Emergency Medicine Physician 2	Humerus	24.0	24.0	0.0	N/A	N/A	N/A
Emergency medicine	Emergency Medicine Physician 2	Radius	16.0	18.2	0.0	N/A	N/A	N/A
Emergency medicine	Emergency Medicine Physician 2	Ulna	36.0	36.0	4.0	N/A	N/A	N/A
Emergency medicine	Emergency Medicine Physician 2	Femur	20.0	20.8	0.0	N/A	N/A	N/A
Emergency medicine	Emergency Medicine Physician 2	Tibia	16.0	16.0	0.0	N/A	N/A	N/A
Emergency medicine	Emergency Medicine Physician 2	Fibula	16.0	16.7	0.0	N/A	N/A	N/A
Emergency medicine	Emergency Medicine Physician 3	Humerus	32.0	33.3	0.0	N/A	N/A	N/A
Emergency medicine	Emergency Medicine Physician 3	Radius	0.0	0.0	8.0	N/A	N/A	N/A
Emergency medicine	Emergency Medicine Physician 3	Ulna	20.0	22.7	4.0	N/A	N/A	N/A
Emergency medicine	Emergency Medicine Physician 3	Femur	48.0	54.5	4.0	N/A	N/A	N/A
Emergency medicine	Emergency Medicine Physician 3	Tibia	12.0	15.0	4.0	N/A	N/A	N/A
Emergency medicine	Emergency Medicine Physician 3	Fibula	8.0	9.5	8.3	N/A	N/A	N/A
VLM	ChatGPT-5.2	Humerus	8.0	33.3	7.7	4.0	30.0	4.0

Group	Reader/Model	Bone	T0 Pipeline	T0 Conditional	T0 Over-explain	T1 Pipeline	T1 Conditional	T1 Over-explain
VLM	ChatGPT-5.2	Radius	8.0	66.7	0.0	4.0	50.0	0.0
VLM	ChatGPT-5.2	Ulna	20.0	83.3	0.0	12.0	70.0	0.0
VLM	ChatGPT-5.2	Femur	20.0	83.3	4.0	12.0	70.0	2.0
VLM	ChatGPT-5.2	Tibia	12.0	100.0	0.0	8.0	80.0	0.0
VLM	ChatGPT-5.2	Fibula	0.0	0.0	4.2	2.0	20.0	2.0
VLM	Gemini 3 Pro	Humerus	4.0	25.0	3.8	2.0	20.0	2.0
VLM	Gemini 3 Pro	Radius	4.0	33.3	4.0	2.0	25.0	2.0
VLM	Gemini 3 Pro	Ulna	20.0	83.3	0.0	12.0	70.0	0.0
VLM	Gemini 3 Pro	Femur	16.0	80.0	8.0	10.0	66.7	4.0
VLM	Gemini 3 Pro	Tibia	8.0	100.0	0.0	6.0	75.0	0.0
VLM	Gemini 3 Pro	Fibula	0.0	0.0	4.2	2.0	20.0	2.0
VLM	Claude Sonnet 4.5	Humerus	4.0	16.7	38.5	2.0	15.0	20.0
VLM	Claude Sonnet 4.5	Radius	8.0	33.3	24.0	4.0	25.0	16.0
VLM	Claude Sonnet 4.5	Ulna	24.0	66.7	16.0	12.0	55.6	10.0
VLM	Claude Sonnet 4.5	Femur	8.0	40.0	36.0	6.0	33.3	24.0
VLM	Claude Sonnet 4.5	Tibia	4.0	50.0	8.0	4.0	40.0	6.0
VLM	Claude Sonnet 4.5	Fibula	0.0	0.0	12.5	2.0	16.7	8.0

Pipeline exact match ("end-to-end") was defined as the proportion of reference-standard fracture-positive cases in which the fracture decision and both subtype attributes (morphology and displacement) were simultaneously correct. Conditional exact match was calculated among true-positive fracture detections only. Over-explanation was defined as providing a fracture subtype description in reference-standard fracture-negative cases. All bone subgroups included 25 fracture-positive and 25 fracture-negative cases (n=50). Percentages are rounded as provided. VLM, vision language model; repeat assessment (T1) was performed for the models only.

Supplementary Table S7. One-month temporal stability of fracture subtype classification metrics across bones

Model	Bone	T0 Pipeline Exact-Match %	T1 Pipeline Exact-Match %	Δ Pipeline	T0 Conditional Exact-Match %	T1 Conditional Exact-Match %	Δ Conditional	T0 Over-explain %	T1 Over-explain %	Δ Over-explain
ChatGPT-5.2	Humerus	8.0	4.0	−4.0	33.3	30.0	−3.3	7.7	4.0	−3.7
	Radius	8.0	4.0	−4.0	66.7	50.0	−16.7	0.0	0.0	0.0
	Ulna	20.0	12.0	−8.0	83.3	70.0	−13.3	0.0	0.0	0.0
	Femur	20.0	12.0	−8.0	83.3	70.0	−13.3	4.0	2.0	−2.0
	Tibia	12.0	8.0	−4.0	100.0	80.0	−20.0	0.0	0.0	0.0
	Fibula	0.0	2.0	+2.0	0.0	20.0	+20.0	4.2	2.0	−2.2
Gemini 3 Pro	Humerus	4.0	2.0	−2.0	25.0	20.0	−5.0	3.8	2.0	−1.8
	Radius	4.0	2.0	−2.0	33.3	25.0	−8.3	4.0	2.0	−2.0
	Ulna	20.0	12.0	−8.0	83.3	70.0	−13.3	0.0	0.0	0.0
	Femur	16.0	10.0	−6.0	80.0	66.7	−13.3	8.0	4.0	−4.0
	Tibia	8.0	6.0	−2.0	100.0	75.0	−25.0	0.0	0.0	0.0
	Fibula	0.0	2.0	+2.0	0.0	20.0	+20.0	4.2	2.0	−2.2
Claude Sonnet 4.5	Humerus	4.0	2.0	−2.0	16.7	15.0	−1.7	38.5	20.0	−18.5
	Radius	8.0	4.0	−4.0	33.3	25.0	−8.3	24.0	16.0	−8.0
	Ulna	24.0	12.0	−12.0	66.7	55.6	−11.1	16.0	10.0	−6.0
	Femur	8.0	6.0	−2.0	40.0	33.3	−6.7	36.0	24.0	−12.0
	Tibia	4.0	4.0	0.0	50.0	40.0	−10.0	8.0	6.0	−2.0
	Fibula	0.0	2.0	+2.0	0.0	16.7	+16.7	12.5	8.0	−4.5

Pipeline Exact-Match: Among all true fracture cases, proportion with correct fracture decision and correct morphology and correct displacement in the same case. Conditional Exact-Match: Among true-positive fracture detections, proportion with correct morphology and displacement. Over-explain: Proportion of fracture-negative cases with unnecessary fracture subtype descriptions. All bones were normalized to n = 50 (25 fracture / 25 non-fracture). Percentages are rounded to one decimal place. Δ = T1 − T0, in percentage points.

Supplementary Figure S1.

Representative screenshot illustrating the standardized prompt, radiographic image submission interface, and output format used for vision language model evaluations. The same prompt structure and output requirements were applied across all models and both time points (T0 and T1). The image file is supplied separately.

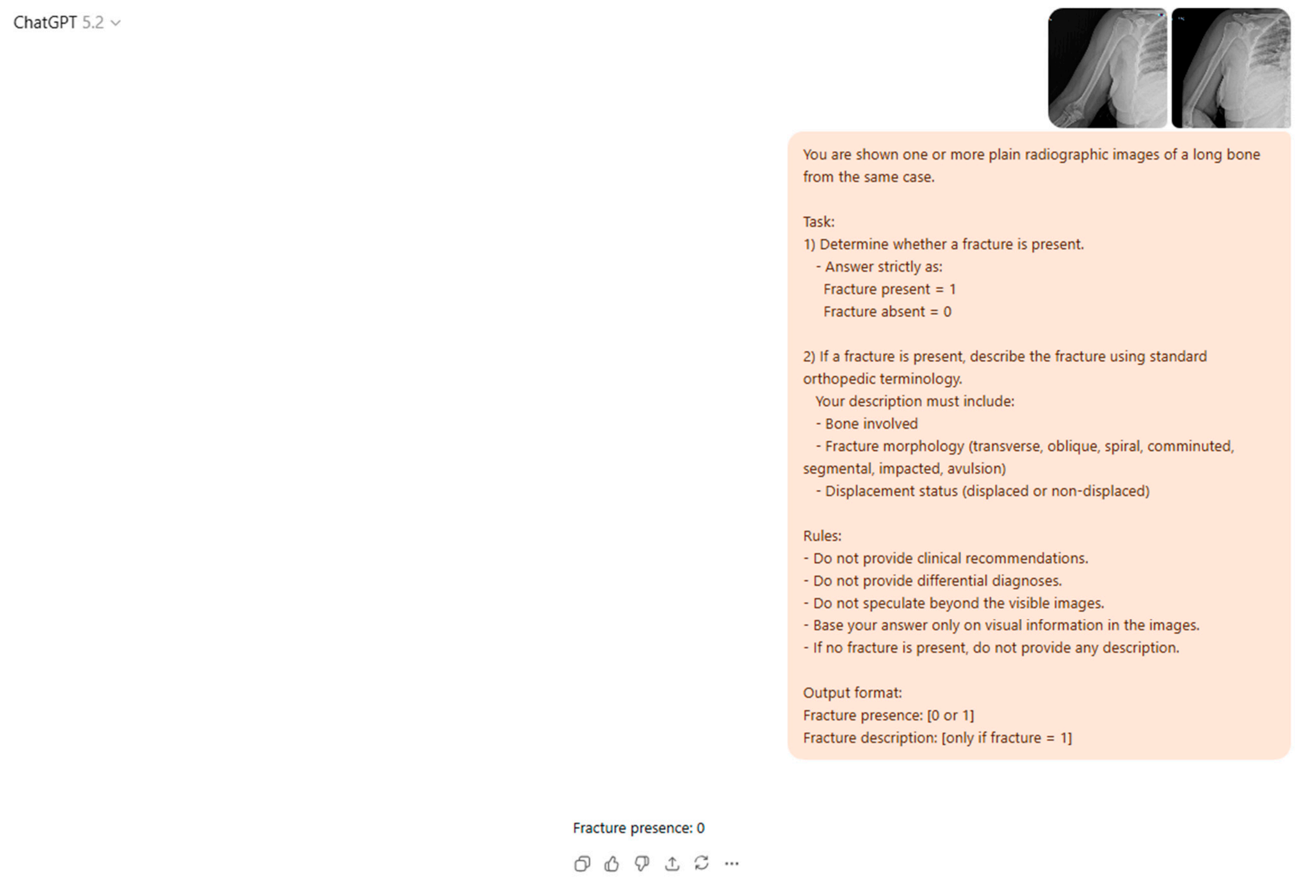

Supplement: Supplementary file 1 [file tomography-12-00113-s001.zip › tomography-4432111-supplementary.pdf]
